# Health system delay in treatment of multidrug resistant tuberculosis patients in Bangladesh

**DOI:** 10.1186/s12879-015-1253-9

**Published:** 2015-11-16

**Authors:** Mahfuza Rifat, John Hall, Christopher Oldmeadow, Ashaque Husain, Abul Hasnat Milton

**Affiliations:** School of Medicine and Public Health, Faculty of Health and Medicine, the University of Newcastle, HMRI Building Lot 1 Kookaburra Circuit, New Lambton Heights, Newcastle, NSW 2305 Australia; BRAC, Dhaka, Bangladesh; National Tuberculosis Control Programme, Directorate General of Health Services, Dhaka, Bangladesh

**Keywords:** Delay, MDR-TB, Multidrug resistant tuberculosis, Barrier to treatment, Health system, Private practitioner

## Abstract

**Background:**

Bangladesh is one of the 27 high burden countries for multidrug resistant tuberculosis listed by the World Health Organization. Delay in multidrug resistant tuberculosis treatment may allow progression of the disease and affect the attempts to curb transmission of drug resistant tuberculosis. The main objective of this study was to investigate the health system delay in multidrug resistant tuberculosis treatment in Bangladesh and to explore the factors related to the delay.

**Methods:**

Information related to the delay was collected as part of a previously conducted case–control study. The current study restricts analysis to patients with multidrug resistant tuberculosis who were diagnosed using rapid diagnostic methods (Xpert MTB/RIF or the line probe assay). Information was collected by face-to-face interviews and through record reviews from all three Government hospitals providing multidrug resistant tuberculosis services, from September 2012 to April 2013. Multivariable regression analysis was performed using Bootstrap variance estimators. Definitions were as follows: Provider delay: time between visiting a provider for first consultation on MDR-TB related symptom to visiting a designated diagnostic centre for testing; Diagnostic delay: time from date of diagnostic sample provided to date of result; Treatment initiation delay: time between the date of diagnosis and date of treatment initiation; Health system delay: time between visiting a provider to start of treatment. Health system delay was derived by adding provider delay, diagnostic delay and treatment initiation delay.

**Results:**

The 207 multidrug resistant tuberculosis patients experienced a health system delay of median 7.1 weeks. The health system delay consists of provider delay (median 4 weeks), diagnostic delay (median 5 days) and treatment initiation delay (median 10 days). Health system delay (Coefficient: 37.7; 95 %; CI 15.0–60.4; p 0.003) was associated with the visit to private practitioners for first consultation.

**Conclusions:**

Diagnosis time for multidrug resistant tuberculosis was fast using the rapid tests. However, some degree of delay was present in treatment initiation, after diagnosis. The most effective way to reduce health system delay would be through strategies such as engaging private practitioners in multidrug resistant tuberculosis control.

## Background

Multidrug resistant tuberculosis (MDR-TB) is a major challenge to worldwide tuberculosis (TB) control [1]. Despite the progress in detection, in 2013 a total of 55 % the estimated MDR-TB were under-detected and 29 % of the diagnosed patients were not on treatment [2]. Delay in TB treatment may result in disease transmission, progression, and poor treatment outcome including increased risk of death [3]. Several studies have reported that delay in TB initiating treatment contributed to development of MDR-TB [4–6].

Bangladesh is one of the high burden countries for TB and has also been listed on the 27 high burden countries for MDR-TB by the World Health Organization (WHO) [2]. Due to the overall high TB burden in Bangladesh, the proportion of patients with MDR-TB (1.4 % and 29 %, among the new and previously treated TB patients, respectively) amounts to 4700 people (2100 and 2600 among new and previously treated TB patients, respectively), which provides a significant challenge for the national tuberculosis control programme [2].

Delay in MDR-TB treatment may cause more suffering to the affected patients as well as hinder the attempts to curb the spread of MDR-TB. Delay in initiation of tuberculosis treatment among the drug sensitive TB patients has been reported in many studies [7]. Some studies reported delay in commencing treatment of MDR-TB patients, although diagnostic and treatment initiation delays were frequently reported in these studies; the context and definition of delays were variable [8–14]. Some studies focused on rapid diagnostic methods for MDR-TB detection, reporting either the time taken for diagnosis or time from diagnosis to treatment initiation [8, 10, 14, 15]. We could only find one study on patient-related delay among the MDR-TB patients and its associated factors, a qualitative study carried out in Cape Town, South Africa [16]. The study reported inaccurate perception of their symptoms as an important factor in the delay in seeking care. Diagnostic delay was inherent in the MDR-TB management procedure while the diagnosis of MDR-TB relied on culture methods, which require longer time than the more advanced tests now routinely in use [17]. In 2012, the National TB control programme of Bangladesh (NTP) adopted rapid tests such as automated real time PCR (Xpert MTB/RIF) and Line probe assays to diagnose MDR-TB/Rifampicin resistant TB (RR-TB) as recommended by the World Health Organization [18, 19]; these methods reduce the time needed for diagnosis.

In Bangladesh, TB service is integrated in the basic health care services and available in all hospitals at sub-district level and below, in chest disease clinics, in district and medical college hospitals and in urban health centres run by government and non-government organizations (NGOs) [20]. Bangladesh has a dynamic NGO sector providing TB control services in collaboration with NTP through a partnership approach [21]. Different types of health care providers administer TB services in Bangladesh, including both qualified private practitioners and informal providers [22, 23]. Private practitioners are popular in Bangladesh irrespective of patients’ income level and residence [24]. However, NTP does not have strong linkage with the private sector. As in many other countries, TB services provided by the private sector are poor, with use of inappropriate treatment and poor case holding, leading to incomplete treatment and drug resistance [24].

Delays in the treatment of drug-sensitive TB patients have been reported in several studies carried out in Bangladesh [3, 25–27]. Our study aims to explore delays related to the commencement of treatment for MDR-TB patients caused by the health system, namely provider delay, diagnostic delay and treatment initiation delay. Further, we aim to explore possible factors related to the health system delay, as well as to offer potential solutions.

## Methods

### Study population and setting

MDR-TB patients were identified as part of a previously conducted case–control study on risk factors associated with MDR-TB in Bangladesh when information related to treatment delay was also collected [28]. The study included 250 MDR-TB and 750 non-MDR-TB tuberculosis patients. In the current study we restrict the analysis to data related to the delay in treatment of MDR-TB patients.

NTP Bangladesh adopted the standardized regimen for treating MDR-TB and started the DOTS Plus project, in 2008 [29]. Currently one hospital at national level and four at regional level are providing MDR-TB diagnosis and treatment services. These hospitals are equipped with reference laboratories and a MDR-TB treatment ward. Presumptive MDR-TB patients who are identified at the district, sub-district or lower level are referred to these central and regional level hospitals for diagnosis and treatment initiation of MDR-TB, according to the national guideline [29].

At the time of our data collection, MDR-TB patients from all over Bangladesh (central, district and subdistrict level) were referred to one of three government hospitals, i.e. the national hospital in Dhaka or a regional hospital in either Chittagong or Rajshahi. All eligible MDR-TB patients from these hospitals who were admitted from September 2012 to mid-April 2013 were recruited. At that time the rapid diagnostic tests were still evolving in Bangladesh, and some of the MDR-TB patients were diagnosed through the conventional culture and DST method. We only included the MDR-TB patients diagnosed by rapid diagnosis tests to ensure valid comparisons.

Except for the diagnostic criteria, the inclusion and exclusion criteria of this study were similar to the previous study [28]. The NTP has adopted automated real time PCR (Xpert MTB/RIF) as the preferred rapid diagnostic tool of MDR-TB patients. Culture and Drug Sensitivity Testing (DST) and Line probe assays were also used by NTP [29]. Xpert MTB/RIF diagnoses only Rifampicin resistance. However, patients who are resistant to Rifampicin are generally also resistant to Isoniazid (another first-line drug). Mono-resistance to Rifampicin is fairly uncommon (0.2 % and 0.4 % among new and previously treated patients, respectively), as shown by a recent drug resistance survey (DRS) conducted in Bangladesh [30]. This study included 207 MDR-TB patients who were diagnosed by the rapid tests, i.e. Xpert MTB/RIF or Line probe assays, to maintain the consistency in overall delay related information. We had excluded 34 patients who were diagnosed by the conventional culture and DST method. Nine patients were excluded due to missing information. The NTP uses a laboratory based in Antwerp, Belgium as quality control for diagnosis of MDR-TB patients.

### Data collection and definitions

**Fig. 1:**
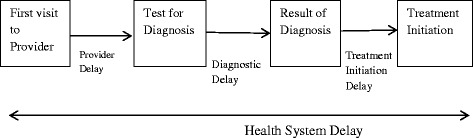
Definition of delays used in this study. Adapted from: Rifat M, Rusen ID, Islam MA, Enarson DA, Ahmed F, Ahmed SM, Karim F: Why are tuberculosis patients not treated earlier? A study of informal health practitioners in Bangladesh. *International Journal of Tuberculosis & Lung Disease* 2011, 15(5):647–651

Definitions used in this study are as follows: Provider delay: time between visiting a provider to visiting designated diagnostic centre for testing; Diagnostic delay: time between the diagnostic sample provided to date the result is available; Treatment initiation delay: time between the date of diagnosis and the date of treatment initiation; Health system delay: time between visiting a provider to start of treatment. Health system delay was derived by adding provider delay, diagnostic delay and treatment initiation delay (Fig. 1). In this study, the term private practitioner refers to clinicians who are at least medical graduates; informal providers are the one without medical degree (MBBS), i.e. village doctors, medical assistants.

We tried to keep the definitions in line with a study conducted in Bangladesh that focused on treatment delay among drug sensitive TB patients due to health care providers [3]. Delay-related definitions used for drug-sensitive TB were not completely applicable to MDR-TB. As it is difficult to estimate patient delay (defined as the time between the onset of symptom to visiting a health care provider) for MDR-TB, as most of the patients had been treated previously, we focused our study on the delay due to the health system. We interviewed patients regarding their first visit to a health provider, assuming the patient would visit a provider when they perceived the symptoms as worsening or suspected a lack of response to their ongoing treatment. The provider delay in our study is based on patients’ perceptions. Most of the participants (97 %) were TB patients who had previously received treatment for TB. We asked the patients specifically how many weeks before the MDR-TB diagnosis they had visited a health provider for their current problem (i.e. worsening of TB symptoms, or feeling that the disease was not responding to treatment).

Trained investigators collected information from the study participants by face-to-face interview using a pre-tested questionnaire, and by review of records. Information related to the time frame of the patient’s visit to the provider was collected through interview, and since their recall may not be accurate, it was expressed in weeks rather than days. The dates of diagnosis and treatment were taken from the hospital records, and the diagnostic and treatment delays were expressed in days.

### Statistical analysis

Socio-demographic characteristics were summarised using means and standard deviations for continuous variables and counts/percentages for categorical variables. Multivariable regression models were used to estimate the effect of age, sex, education, occupation, residence status, type of TB the patient, and the provider visited for first consultation, on health system delay. We have included patient characteristics such as education, occupation, residence status and income in the multivariable analysis, assuming that these characteristics may influence the choice of provider and affect the health system delay. Since the distribution of delay was skewed, we used Bootstrap variance estimators for regression analysis. Regression coefficients are presented with 95 % confidence intervals and associated p values for simple and Omnibus hypotheses. Data analysis was carried out using Stata statistical software version 12 (StataCorp LP, TX, USA).

### Limitation of the study

This study includes only patients who were already enrolled in MDR-TB treatment. We do not have information about patients who did not start treatment after diagnosis. Unlike with the delay in drug-sensitive TB patients, it is difficult to determine the date for the onset of symptoms that prompted a visit to a clinician among the MDR-TB patients. Information related to provider delay was based on patients’ information recalled during face-to-face interviews, and could not be verified.

### Ethics considerations

The study was approved by the Human Research Ethics Committee (HREC) of the University of Newcastle (UoN), Australia, and the Bangladesh Medical Research Council (BMRC), Dhaka, Bangladesh. An information sheet describing the purpose of the study and the individuals’ rights as study participants was handed to each participant to read. For individuals with inadequate literacy, the information sheet was read out by the interviewers. Participants consented by signing the consent form, or if unable to do so, by adding their thumb impression. All patients had been treated through NTP Bangladesh.

## Results

Socio-demographic characteristics of the patients are presented in Table 1. The median delay caused by health system factors was 7.1 weeks. Provider delay (median 4 weeks), diagnostic delay (median 5 days) and treatment initiation delay (median 10 days) make up the health system delay (Table 2).

**Table 1:** Socio-demographic and clinical characteristics of the multidrug resistant tuberculosis (MDR-TB) patients in Bangladesh

Variables	Rapid tests (*n* = 207)
	n (%)
Gender	
Male	138 (66.7)
Female	69 (33.3)
Education	
None	53 (22.0)
Secondary and below	169 (70.1)
Higher secondary and above	19 (7.9)
Provider visited for first consultation	
DOTS	91 (45.4)
Private	89 (43.8)
Informal	22 (10.8)
Residence status	
Rural	102 (49.3)
Urban	105 (50.7)
Occupation	
None	8 (3.9)
Service	66 (31.9)
Farmer	16 (7.7)
Student	5 (2.4)
Homemaker	39 (18.8)
Factory worker	16 (7.7)
Business	36 (17.4)
Self employed	12 (5.8)
Transport worker	9 (4.4)
Smoking status	
Smoker	103 (49.8)
Non- smoker	104 (50.2)
Type of TB patient	
New	5 (2.4)
Previously treated	202 (97.6)
Treatment outcome of previously treated patients	
Cured	16 (7.9)
Completed	37 (18.3)
Default from treatment	5 (2.5)
Treatment failure	119 (58.9)
No record available	25 (12.4)
Age (mean ± SD)	34 ± 12.0
Income (mean ± SD)	13664.3 ± 11521.7

Age and Income are expressed in the table is the mean ± Standard Deviation. All other variables are expressed as n (%)

Income is in Bangladeshi taka (BDT), monthly. 1 USD =78 BDT approximately

DOTS: National TB control programme designated centres for TB treatment

**Table 2:** Delays in treatment among the multidrug resistant tuberculosis (MDR-TB) patients of Bangladesh (*n* = 207)

	Median	IQR	Mean	SD
Health System delay (weeks)	7.1	8.6 (4.6–13.3)	10.5	11.25
Provider delay (weeks)	4	6 (2–8)	6.8	9.6
Diagnostic delay (days)	5	6 (1–7)	5.9	8.1
Treatment initiation delay (days)	10	17 (6–23)	20.5	28.9

Health system delay includes the provider delay, diagnostic delay and treatment initiation delay

Diagnostic delay includes the patients who had the rapid tests such as Gene Xpert and or LPA

Diagnostic delay, provider delay and health system delay had 3, 4 and 7 missing values, respectively

Standard deviation (SD), Interquartile range (IQR)

Total 89 MDR-TB patients (43.8 %) consulted a private practitioner first, for their MDR-TB related symptom (Table 1), which included 86 previously treated TB patients. Only 9.5 % of the previously treated TB patients had been treated in the private sector for their previous TB disease and the rest (90.5 %) were treated in a DOTS centre (not shown in table).

Multivariable regression analysis (Table 3) of health system and associated factors causing delay in the treatment of MDR-TB patients are shown in Table 3. Patients who visiting private practitioners for the first consultation experienced a greater health system delay compared to those visiting the NTP designated DOTS centre (mean difference, 37.7 days; 95 % CI 15.0–60.4; p 0.003).

**Table 3:** Factors related to health system delay of multidrug resistant tuberculosis (MDR-TB) patients of Bangladesh

		Univariate analysis (*n* = 200)				Multivariable analysis (*n* = 200)				
Variable	Median Health system delay	Coefficient^a^	p*	95 % Confidence Interval		Coefficient^a^	p*	p**	95 % Confidence Interval	
				Lower	Upper				Lower	Upper
Gender										
Male	51.5	Reference				Reference				
Female	45	−21.9	0.01	−38.9	−4.9	−18.1	0.27		−50.4	14.2
Education								0.35		
No education	43	Reference				Reference				
Up to secondary level	50.5	4.1	0.70	−16.9	25.0	3.5	0.76		−18.7	25.7
Higher secondary and above	54.5	58.0	0.13	−17.6	133.5	58.1	0.15		−20.3	136.5
Provider visited								0.003		
DOTS centre^c^	45	Reference				Reference				
Private practitioners	53	33.6	0.01	10.2	57.0	37.7	0.001		15.0	60.4
Informal provider	59.5	23.4	0.12	−5.9	52.7	26.2	0.13		−8.0	60.5
Residence status										
Rural	51	Reference				Reference				
Urban	46	−1.3	0.89	−19.0	16.4	4.6	0.70		−19.1	28.2
Type of the patients								0.07		
New TB patient	57	Reference				Reference				
Cured, previously treated	45	−4.2	0.83	−41.4	33.0	21.8	0.40		−29.3	72.9
Completed, previously treated	40.5	2.0	0.90	−30.1	34.0	16.5	0.54		−36.5	69.4
Default, previously treated	51	2.0	0.91	−31.1	35.1	42.6	0.16		−16.7	101.9
Failure, previously treated	54	27.4	0.14	−9.3	64.1	51.7	0.08		−5.3	108.7
No outcome recorded, previously treated	56.5	5.1	0.79	−31.3	41.4	23.9	0.42		−34.0	81.7
Occupation								0.66		
None	41.5	Reference				Reference				
Service	51	34.7	0.02	5.4	64.0	17.1	0.34		−17.9	52.1
Farmer	54	25.0	0.10	−4.8	54.8	32.8	0.16		−12.8	78.4
Student	27	−15.3	0.14	−35.6	5.0	−21.7	0.56		−93.9	50.6
Homemaker	49	14.4	0.25	−9.9	38.7	26.2	0.12		−6.8	59.1
Factory worker	54	41.6	0.15	−15.0	98.2	36.6	0.32		−35.1	108.2
Business	48.5	31.8	0.03	2.7	60.8	8.8	0.72		−38.6	56.2
Self employed	70.5	43.9	0.07	−3.5	91.3	45.9	0.13		−13.7	105.4
Transport worker	45.5	51.0	0.24	−33.8	135.8	45.1	0.31		−41.7	131.8
Age (years)	-	−0.02	0.95	−0.5	0.5	−0.3	0.36		−1.0	0.4
Income (BDT)^b^	-	0.001	0.21	−0.001	0.003	0.0003	0.71		−0.001	0.002

^a^Regression coefficients reflect adjusted difference in mean delay (days), while all other factors remain constant

^b^Income is in Bangladeshi taka (BDT), monthly. 1 USD =78 BDT approximately

^c^DOTS centres are the NTP designated treatment centres for tuberculosis

p*is the value that the regression coefficient is zero

p**is the value form the omnibus test that all coefficients for that variable are zero

## Discussion

Our study found that time taken for diagnosis of MDR-TB is five days since the introduction of rapid tests in the programme; subsequently it took ten days to initiate treatment. A recent study on pre-diagnosis and pre-treatment attrition of MDR-TB patients of Bangladesh presented the median time for diagnosis and treatment initiation as four and five days, respectively [31]. The study included 163 MDR-TB patients diagnosed by Xpert MTB/RIF from selective areas which are supported by one NGO (BRAC) in Bangladesh. Whereas, our study included patients from all three government hospitals providing MDR-TB services at that time, which included patients referred from all areas of Bangladesh (including the area not supported by BRAC), during the study period. Laboratory turnaround time reported by another study conducted in Cape Town, South Africa was less than one day using Xpert based algorithm and 24 days using Line probe assay based algorithm [10]. The study also reported time to initiate treatment after diagnosis as 10 and 14 days in Xpert and Line probe assay based algorithm, respectively [10]. Similar results were found in a multicounty study which reported median time to detect rifampicin resistant as one day for Xpert MTB/RIF test and 20 days for Line Probe Assay based test [8]. Another study on MTB-DR Plus showed reduction in laboratory processing time (median 22 days) compared to culture based DST which was 55 days; whereas it took 20 days of operational delay to start the treatment [14]. Diagnosis time using MTB-DR plus was also reported as 4.2 and 11 days in Georgia and India, respectively [15, 32]. In our study, time needed for diagnosis is satisfactory. However, there was a remarkable delay in treatment initiation before the diagnosis, which was also observed in other studies on rapid tests for MDR-TB diagnosis [10, 14].

Unlike most other studies on the delay to treatment of MDR-TB patients, the definition of health system delay in our study includes the delay related to the visit to the health care provider. Median health system delay is 7.1 weeks in our study, which is mainly due to provider delay (4 weeks). Those patients who visited a private practitioner after perceiving their symptom during their current MDR-TB episode, experienced longer health system delays than patients who visited a NTP designated DOTS centre. In another study on drug sensitive TB patients in Bangladesh, visiting informal providers was associated with longer health system delay [3]. In contrast, we did not find any association between delay in treatment and visiting informal providers. MDR-TB patients may prefer qualified practitioners to informal providers as treatment is more complicated. We also found that many of the MDR-TB patients, who consulted private practitioners first for their current problem, had been treated in DOTS centre during previous TB treatment and this group of patients were reliant on private practitioners for their current complicated problem. This finding indicates that the MDR-TB patients might have visited multiple providers during the course of previous and current TB disease. However our finding concludes those who had in touch with private practitioners had experienced greater health system delay.

The provider delay may be due to lack of awareness of referral services by private practitioners. A sputum result of drug-sensitive TB patients at 5^th^ month or 8^th^ month of treatment forms the basis for a decision on referral for MDR-TB diagnosis, according to the national guideline. Waiting for the scheduled sputum conversion result could be another factor for provider delay. However, the national guideline also allows clinicians to refer a patient for MDR-TB screening [29]. NTP and its NGO partners in Bangladesh are involved in linking the private practitioners to the national TB control programme [24], encouraging private practitioners to refer TB patients to NTP designated DOTS centres where they receive tuberculosis treatment free of charge. However, many of the private practitioners who are not linked to the NTP often do not treat the TB patients according to the International Standards for Tuberculosis Care (ISTC) [24, 33, 34]. Strengthening the involvement of private practitioners in TB control with emphasis on MDR-TB is needed.

Delay in treatment initiation of MDR-TB, was also reported in a few other studies based on conventional DST methods. Two studies using conventional culture and DST for MDR-TB diagnosis reported a total time from diagnosis to treatment initiation of 12.4 weeks and 17 weeks in Kwazulu Natal, South Africa and Cameroon, respectively [11, 13]. Time for diagnosis and treatment initiation using conventional culture was 246 to 283 days, respectively among children, if the information of their MDR-TB contact was not one of the criteria for diagnosis [12]. Time taken at different stages of MDR-TB management using conventional culture and DST method, starting from sample collection to start of treatment, was also reported in another study which presents a total turnaround time of 5 months which was almost double of the bacteriological procedure [35]. To get a patient started on treatment after diagnosis took 12.8 days in Vietnam [9].

Unnecessary delays should be prevented to control further transmission of MDR-TB. The delay between diagnosis and initiating treatment might be due to the need for other necessary medical examinations such as clinical tests prior to initiation of treatment (e.g. liver function test, Xray, thyroid profile, blood sugar) [10]. Other operational issues adding to the delay might be sample transportation, laboratory-based diagnostic and patient notification, and admission to hospital, or may be due to the protocol for processing smear-negative samples [14, 35].

The reason for the delay in treatment initiation in Bangladesh could be due to the initial hospitalization requirement for MDR-TB treatment, according to the protocol that was usually 6 to 8 months. MDR-TB patients may require preparation time to get admitted to hospital for months. Again, those hospitals also had bed limitation to enrol all patients at a time and patient has to wait in queue. Need for shortening of hospital stay was felt by the programme to make a balance between the number of patients diagnosed by rapid tests and the number of beds available at MDR-TB hospitals.

A pilot project on shortening hospital stay is under way, i.e. to start ambulatory treatment after two consecutive negative sputum culture results. Patients were treated as ambulatory at community level after initial hospitalization. Ambulatory or community-based treatment for MDR-TB is also recommended by the WHO, whenever possible [2]. One study reported that the reason for treatment initiation delay was due to the need for a decision made by the Programmatic Management of Drug Resistant TB (PMDT) Council, and the time taken to assign the patient to the treatment support during the long preparation phase [9]. Another study reported that having a town address was associated with less delay among MDR-TB and we did not find any relationship with urban–rural status and time to treatment, in our study [14].

## Conclusion

Introduction of rapid diagnostic methods have satisfactory time needed for MDR-TB diagnosis. Treatment initiation subsequent to diagnosis was delayed may be due to programmatic factors. This could be improved by identifying specific problems of implementation at programme level. Engaging private practitioners in national MDR-TB control programme should be enhanced to reduce the overall delay in MDR-TB management.

## Abbreviations

DRS: Drug resistant surveillance
DST: Drug-susceptibility testing
ISTC: International standards for tuberculosis care
LPA: Line-probe assay
MDR-TB: Multidrug-resistant tuberculosis
NGO: Non-government organization
NTP: National tuberculosis control programme
PCR: Polymerase chain reaction
PMDT: Programmatic management of drug resistant tuberculosis
RR-TB: Rifampicin-resistant tuberculosis
TB: Tuberculosis
Xpert: Xpert MTB/ RIF
